# The early development of infant siblings of children with autism spectrum disorder: Characteristics of sibling interactions

**DOI:** 10.1371/journal.pone.0193367

**Published:** 2018-03-15

**Authors:** Chloè Bontinck, Petra Warreyn, Sara Van der Paelt, Ellen Demurie, Herbert Roeyers

**Affiliations:** Department of Experimental Clinical and Health Psychology, Research Group Developmental Disorders, Ghent University, Ghent, Belgium; University of Kansas School of Medicine, UNITED STATES

## Abstract

Although sibling interactions play an important role in children’s early development, they are rarely studied in very young children with an older brother or sister with autism spectrum disorder (ASD). This study used a naturalistic, observational method to compare interactions between 18-month-old infants and their older sibling with ASD (*n* = 22) with a control group of 18-month-old infants and their typically developing (TD) older sibling (*n* = 29). In addition, role (a)symmetry and the influence of gender were evaluated. Sibling interactions in ASD-dyads were characterized by higher levels of negativity. Although somewhat less pronounced in ASD-dyads, role asymmetry was present in both groups, with the older child taking the dominant position. Finally, siblings pairs with an older sister were characterized by more positive behaviours. Since differences in sibling interactions may alter the developmental trajectories of both siblings, these early relationships should be taken into account in future ASD research and interventions.

## Introduction

In early development, the social world mainly consists of interactions with caregivers and siblings [1]. Sibling interactions are long-term, intensive relationships that influence the development of both interaction partners [2,3]. Furthermore, they have a unique socialization function during infancy and childhood [4,5]. Positive, nurturing sibling interactions can facilitate social behaviour and relationships. They promote the development of children’s understanding of others’ emotions and thoughts as well as their social competence [6–8]. Children who experience their sibling relationships as harmonious rather than conflictual report higher levels of academic and social competence and lower levels of internalizing and externalizing problems [9]. Along with positive interactions, sibling interactions usually entail a certain level of conflict. Since conflict helps children to learn to manage their anger and stimulates them in finding ways to resolve their quarrels, the presence of conflict combined with positive interactions/nurturance can promote their development [10].

The nature of sibling relationships partially depends on the gender of both interaction partners and gender differences in relationship quality have been reported. Sister pairs are more intimate and harmonious than brother pairs [9,11]. In addition, research shows that boys experience higher levels of sibling rivalry in interaction with a brother or sister (either older or younger) and that dyads in which the older sibling is a boy are more conflictual [9,12].

Sibling relationships are characterized by both complementary and reciprocal interactions [6,13]. Complementary interactions are hierarchical and imply that one interaction partner dominates the other due to greater power or more advanced cognitive skills (because of a difference in age or experience). During reciprocal interactions, both interaction partners have an equal position [6,14]. Both types of interaction stimulate development. While complementary interactions promote the development of more refined social skills, reciprocal interactions enhance the feeling of emotional support and help siblings develop shared meanings [15,16]. Harrist and colleagues [6] found that, in sibling interactions including very young children, both types of interaction are equally represented. With regard to complementary interactions, in typically developing sibling pairs older siblings often take the more dominant position, which is expressed by higher levels of both prosocial and agonistic initiations. In this case, younger siblings maintain the interaction and reinforce the older child’s leadership position by responding positively to positive behaviour and by submitting to negative behaviour [1,6,17,18]. This asymmetric pattern is stable throughout early and middle childhood and is already visible during interactions between preschool-aged children and their younger infant sibling [1]. Reciprocal interactions are mainly characterized by playing together and sharing mutual goals/interests (with verbal/nonverbal communication) [6]. However, due to the lower levels of social engagement, social interest/motivation and reciprocity reported in children with ASD [19–22], both reciprocal and complementary interactions might be compromised in dyads including a child with ASD.

Deficits in social communication and social interaction are among the core features of autism spectrum disorder (ASD) [23]. Research also shows that, in comparison with typically developing controls, siblings of individuals with ASD have an increased risk of developing ASD themselves [24]. In addition, they are at higher risk of showing subclinical features of ASD, referred to as the Broader Autism Phenotype (BAP) [25–27]. During the first years of life, siblings of children with ASD may show atypicalities in their cognitive, motor, language, and/or social development [28–30]. This increased recurrence rate suggests that genes are involved and studies show that the contribution of genetic factors to the development of ASD and the BAP is substantial, with at least a moderate genetic heritability (e.g., [31]). However, genetics cannot account for all the variability found in (siblings of) children with ASD. Environmental factors such as prenatal, perinatal and/or postnatal factors also contribute to the development of ASD [32–36]. Gene-environment interactions are at the root of both typical and atypical development. Identical genotypes can result in different behavioural outcomes depending on differences in children’s physical or social environment (e.g., life experiences, child rearing practices) [32,37,38].

As sibling interactions are an important aspect of the early social environment, they need to be considered when looking at the development of younger siblings of children with ASD (*hereafter high-risk (HR) siblings)* and their increased risk of ASD/BAP [39]. If and how children with ASD affect the development of their younger sibling also partly depends on characteristics of the HR-sibs. Compared to HR-sibs who show characteristics of BAP/ASD themselves, typically developing HR-sibs might be more resilient or flexible in light of conflict or they might compensate for the social-communicative deficits of their older sibling with ASD [40]. Also, in accordance with the diathesis-stress model, lower quality sibling interactions might interact with pre-existing vulnerabilities such as characteristics of the BAP/ASD, impacting on the development of HR-sibs [41,42]. For example, a lack of positive social exchanges during the sibling interaction may lead to fewer learning opportunities for HR-sibs and a decrease in social input. Furthermore, in line with the theory of observational learning, siblings shape the relationship with each other by observing and imitating one another [43–45]. Accordingly, younger siblings of children with ASD may imitate certain ASD-specific behaviours of their older brother/sister, possibly resulting in behaviours that resemble ASD or the broader phenotype. Imitation also requires interactional synchrony (i.e., the coordination of behaviours and/or emotions between interaction partners in both form and timing), which depends on mutual attention engagement, turn taking and reciprocity between partners [45,46]. The ability to coordinate one’s own behaviour/emotions with those of another person might be compromised in children with ASD or in HR-sibs, negatively influencing the process of observational learning or imitation.

In the current study, the interactional processes involved in dyads including a child with ASD and a younger infant sibling will be explored as a first step to identify the importance of sibling interactions in families with a child with ASD. Existing studies on sibling interactions in children with ASD are rare. In an observational study, Knott and colleagues [47] compared sibling relationships in pairs including a child with ASD (mean age: 6 years) and their younger/older sibling (mean age: 6.6 years) with pairs including a child with Down Syndrome (DS) (mean age: 5.2 years) and their younger/older sibling (mean age: 5.6 years). They found that children with ASD spent less time with their sibling, initiated the interaction less frequently and showed less variation in their prosocial behaviour than children with DS. Furthermore, children with DS were more responsive to their siblings’ positive initiations, than children with ASD. Using parent report, Walton and Ingersoll [48] found that, in interaction with their brother/sister with ASD (mean age: 9.35 years), typically developing siblings (mean age: 10.43 years) showed lower levels of involvement and higher levels of avoidance compared to siblings of typically developing children. Based on child report, Kaminsky and Dewey [49] concluded that siblings (mean age: 11.67 years) of children with ASD (mean age: 9.79) report less conflict with their sibling than siblings of typically developing children. Regarding role (a)symmetry, the studies of Knott and colleagues [40,47] showed that in dyads with a child with ASD, the younger siblings (without ASD) initiate the interaction more often, taking over the dominant position of the oldest child with ASD and at the same time stimulating communication in children with ASD.

The previous studies report meaningful findings in school-aged children. However, early social-communicative deficits in children who later develop BAP/ASD are already visible in the second year of life [28,50]. Consequently, sibling interactions between children with ASD and their *infant* siblings need to be considered as well. For example, HR-sibs are at increased risk of showing deficits in early social-communicative competencies (e.g., joint engagement, imitation, gestures). This could translate into differences in social engagement, imitation and sharing during sibling interactions, which may in turn impact on the outcome of HR-sibs. Although several studies evaluated the early development of HR-sibs (e.g., [51,52]), we are the first to consider the potential role that early sibling interactions can play in the expression of BAP or ASD in HR-sibs. The assessment of sibling interactions can shed light on the pathways between early vulnerabilities and later outcome [39].

In addition to the focus on older children, there is a shortage of naturalistic observations. Sibling interactions have predominantly been studied by means of questionnaires (e.g., [48,49,53]), which have some disadvantages such as rater bias in parents [54,55]. Child report also requires that children are old enough to report about their experiences. In addition, there is an important difference between reporting about sibling relations in terms of cognitions and/or perceptions and observing the specific dynamics of these interactions. These disadvantages can be avoided by using a naturalistic, observational method, which can also provide a broader picture [56–58].

The present study aimed to evaluate three important aspects of sibling interactions: 1) The interactive behaviour of both siblings; 2) Role (a)symmetries; 3) The effect of gender. To this end, we observed sibling interactions in dyads including 18-month-old infants and their older sibling with ASD as well as in typically developing control dyads in a naturalistic, familiar setting (i.e., the children’s home). First, sibling interactions were compared between groups to identify possible differences between dyads including a child with ASD and typically developing dyads. Based on previous studies [40,47,48], lower levels of interactive behaviour were expected in dyads with children with ASD. Next, role (a)symmetry was evaluated for each group separately. To this end, the behaviour of the younger and older sibling within each group was compared in terms of positive and negative social initiations and responses. In line with previous research, we expected clear role asymmetry in the control group with the older child assuming a more dominant position [1,17,18]. Contrary to the findings of Knott and colleagues [40,47] that HR-sibs act as leaders instead of the children with ASD, we did not expect a clear role asymmetry in dyads with a child with ASD. First, given their young age, HR-sibs’ social-communicative abilities are not yet fully developed. Second, children with ASD (and at least a proportion of the HR-sibs) experience social-communicative difficulties. Therefore it is possible that both children lacked the abilities to act as a leader.

As a second research goal, the influence of gender of the older child on the quality of the sibling relationship was explored in the control group. In line with previous research, we expected dyads with an older sister to be more positive than dyads with an older brother [9,11,12].

## Method

All procedures performed in this study were in accordance with the ethical standards of the institutional research committee and with the 1964 Helsinki declaration and its later amendments or comparable ethical standards. This study was approved by the Ghent University Ethical Commission (Faculty of Psychology and Educational Sciences) and informed consent was obtained from all individual participants included in the study.

### Participants

Fifty-one sibling pairs participated in the study (recruited between 2014–2016). All participants were recruited from an ongoing prospective follow-up study of younger siblings of children with ASD, considered to be at increased risk for developing ASD (high-risk siblings; HR-sibs), and a typically developing control group at Ghent University. Twenty-two sibling pairs were high-risk (HR) dyads with an older child with ASD (hereafter, ASD-sib). Nine HR-sibs had one older sibling and another nine had two older siblings. Four HR-sibs had three or more older siblings and two HR-sibs had two siblings with ASD. The HR group consisted of 10 male-male, 9 female-male, 1 male-female and 2 female-female dyads (younger-older). Twenty-nine sibling pairs were low-risk (LR) dyads with a typically developing older child (typically developing sibling; TD-sib) and their younger sibling (low-risk sibling; LR-sib). The LR group had a family history without first-degree relatives with ASD. All LR-sibs had at least one older sibling and four had two older siblings. The LR group consisted of 7 male-male, 7 female-male, 11 male-female and 4 female-female dyads (younger-older). More HR- than LR-sibs had more than one older sibling (59% vs. 14%; χ^2^ = 11.55, *p* = .001).

ASD-sibs received their ASD diagnosis after evaluation by a multidisciplinary team, including assessment of cognitive and social-communicative functioning. Diagnostic status of the older child in each group was confirmed using the Social Responsiveness Scale, Second Edition (SRS-2; [59], Dutch translation by [60]), and the Social Communication Questionnaire (SCQ; [61], Dutch translation by [62]). In the HR group, the SCQ and SRS were available for all 22 children/ASD-sibs. 12 children scored above the threshold for ASD on both the SCQ (threshold = 15) and the SRS (threshold = 48), the other ten scored above the threshold on the SRS. In the LR group, SRS and SCQ were available for 25 and 26 children/TD-sibs, respectively. All scored below the ASD threshold on the SCQ, and all but one (total score of 69) scored below the ASD threshold on the SRS. Since further evaluation of this child (SCQ score, parent information/concerns) revealed no reasons to suspect ASD, we decided to include this dyad in further analyses. SCQ and SRS scores are presented in Table 1. Cognitive functioning of children with ASD was assessed using either the Wechsler Intelligence Scale for Children (WISC-III-NL; [63]), the Wechsler Preschool and Primary Scale of Intelligence (WPPSI-III-NL; [64]), the Snijders-Oomen Non-Verbal Intelligence Test (SON-R; [65]), or the Bayley Scales of Infant Development (BSID-II-NL: [66]; Bayley-III-NL: [67]). One child with ASD had a total intelligence quotient or developmental index that was very low (IQ<55), eight children scored below average (IQ<85), and eleven children scored in the normal range (IQ between 85–115). For two children, information on cognitive functioning was not available.

**Table 1:** Sample characteristics.

	Low-risk (*n* = 29)	High-risk (*n* = 22)	
	Younger sibling		
Chronological age			
*M(sd)*	18.37 (.54)	18.52 (.85)	*F*(1,49) = .59
Range	17.17–19.33	17.37–20.43	
Sex ratio (M:F)	18:11	11:11	*χ*^*2*^(1) = .74
Developmental level (14 months)			
*M(sd)*	104.85 (9.29)	97.94 (11.86)	*F*(1,43) = 4.78*
Range	92.00–126.00	79.00–120.00	
	Older sibling		
Chronological age			
*M(sd)*	52.61 (14.85)	89.43 (39.10)	*U* = 107.50***
Range	32.97–90.30	46.00–186.07	
Sex ratio (M:F)	14:15	19:3	*χ*^*2*^(1) = 7.95**
Social Communication Questionnaire (SCQ)			
*M(sd)*	3.15 (2.43)	17.23 (7.03)	*F*(1,46) = 91.602***
Range	0.00–8.00	6.00–30.00	
Social Responsiveness Scale (SRS)			
*M(sd)*	24.96 (13.54)	102.59 (27.78)	*F*(1,48) = 199.721***
Range	3.00–69.00	61.00–152.00	
	Sibling pair		
Family SES (*M(sd)*)	51.79 (6.96)	40.77 (12.28)	*U* = 151.50**
Time spent together (%)			*χ*^*2*^(1) = 5.71
*Never/seldom*	3,4%	22,7%	
*Sometimes*	34,5%	40,9%	
*Often/always*	62,1%	36,3%	
Daycare attendance (%)	93%	64%	*χ*^*2*^(1) = 6.89*

*Note*. Chronological age is reported in months

*p<.05

**p<.01

***p<.001

Sample characteristics are presented in Table 1. With regard to the younger sibling, no differences were found in chronological age or sex ratio. As all younger siblings participated in the prospective follow-up study, information on their developmental level at the age of 14 months was available, as tested with the Mullen Scales of Early Learning (MSEL; [68]). The average developmental quotient (DQ) was higher in the LR group (*F*(1,43) = 4.78, *p* = .034). Concerning the older siblings, ASD-sibs were older than TD-sibs (*U* = 107.50, *p*<.001) and there was a significant difference in sex ratio, with an overrepresentation of boys in ASD-sibs (*χ^2^*(1) = 7.95, *p* = .007).

The families’ socioeconomic status (SES) was calculated using Hollingshead’s four factor index [69]. LR families had an average social status of 51.79 (range: 34.50-66.00) and HR families had an average social status of 40.77 (range: 22.00-66.00). The difference between groups was significant (*U* = 151.50, *p* = .001).

Additional information was collected with regard to the amount of time children spent playing together (seldom/sometimes/often) and to what extent both children attended school or day care centres. There were no significant differences between groups in time both children spent together while at home (*χ*^*2*^(1) = 5.71, *p* = .063). However, LR-sibs more frequently went to day care centres than HR-sibs (93% vs. 64%; *χ*^2^ = 6.89, *p* = .013). All older siblings attended school.

### Procedure

Sibling interactions were observed during a short play observation in a familiar context. In all but one cases, this was in the families’ home. One play observation took place in the house of the grandparents. The children were offered a fixed set of toys, namely zoo-themed building blocks, a marble run and an animal sound keyboard, with which they could play consecutively for 10, 10 and 5 minutes, respectively. Before offering a new toy, the previous toy was removed by the researcher. To facilitate the transition between each toy set, children were asked to help clear away the toys or the parent joined briefly to interact with the children. Children were encouraged to play together during the introduction of each toy set and they were given a short verbal instruction (“you can play together with these toys”). Once the children started playing, the researcher observed the play session from the background in order to observe spontaneous behaviour as much as possible. Different sets of toys were chosen to elicit different kinds of play. During play with blocks, the opportunities for parallel play (children play next to each other without interacting) were highest and opportunities for associative (children share or exchange toys, but there is no common goal and interaction is limited) or cooperative (children play together and they have a common goal, work together, make rules, etc.) play were limited. While playing with the marble run children had to share some objects (e.g., the marbles), but they were still able to play parallel as well. Therefore, opportunities for associative and cooperative play were higher during play with the marble run. Finally, during play with the keyboard opportunities for parallel play were limited since there was only one keyboard. By only providing one toy (i.e., limited resources), associative or cooperative play was encouraged. The more children had to share, the higher the risk of conflict.

To avoid unnecessary distractions, parents were asked to switch off all electronic devices (television, tablet) and to remove other toys as much as possible. At the beginning of each play session, parents received general information about the study and were asked to sign an informed consent. During the observation, one parent was always present in the room, continuing normal routines (e.g., household tasks or work).

#### Coding procedure

All play sessions were videotaped and coded afterwards using The Observer XT, version 11.5 [70]. The coding scheme developed for the current study was based on previous work by Abramovitch et al. [71], Knott et al. [47], and Roeyers [22], but further adapted and elaborated. Interactive behaviour was coded in terms of social initiations and responses. *Social initiations* are communicative attempts to initiate a new interaction, directed towards another individual. *Responses* are related to and follow a previous initiation within five seconds. Initiations and responses can be either positive/prosocial (e.g., sharing a toy, allowing the other sibling to do something) or negative (e.g., refusing a request). The absence of a response was also coded (*no response*). In addition to the initiations and responses, the time children spent in interaction with each other (*mutuality*), with the *parent* and with the *experimenter* was also coded. To account for the time not spent in interaction with another person, non-interactive behaviours were coded as well. The following behaviours were included in the present study: *orientation towards the sibling* (without interacting), time spent in a *purposeful activity* (e.g., play), *distressed behaviour* (e.g., anger tantrum, crying), *stereotypical/sensory behaviour*, and *doing nothing/looking at something random*.

Clips were rated by trained master students blind to participant information. 15% of the clips were randomly selected to determine interrater reliability and were coded by all coders. Intraclass correlation coefficients (ICC) were calculated for both the younger and the older child and for each play context. ICC’s ranged between .80 and .99 for the marble run and blocks, and between .78 and .97 for the keyboard. The behaviours ‘no response’, ‘looking at random’ ‘distress’ and ‘stereotypical/sensory behaviour’ were rarely coded, making reliability analysis impossible. Therefore, these behaviours were excluded from further analysis.

### Data analysis

Potential outliers were detected using box plots and visual inspection of the data. Values higher/lower than the mean +/- 3 times the standard deviation (sd) were considered outliers. Assuming that extreme data were not random deviations but characteristic of the sample (e.g., high levels of initiations in high-functioning girls), outliers were replaced by the highest/lowest value allowed (mean +/- 3sd) rather than deleted.

Parametric group comparisons were not possible due to a lack of normal distribution in our data. Consequently, hierarchical regression analyses were used to analyse the degree to which the group status (high-risk vs. low-risk) predicted sibling interaction characteristics. Accordingly, at the first step of the regression model, group was added as a predictor of the sibling interaction variables (positive/negative initiations/responses, mutuality, orientation to sibling). Since the LR and HR group differed in terms of sex ratio and age of the older child, family SES, and developmental level of the younger child, these sample characteristics were added at step 2 of the regression model to evaluate whether sample characteristics changed the possible association between group status and sibling interactions. In addition, non-parametric analyses were used to analyse between-group differences (low-risk vs. high-risk; Mann-Whitney U) and within-group differences (younger vs. older; Wilcoxon Signed-Rank).

To control for the inflation of the Type I error rate due to multiple comparisons, the Bonferroni correction was applied. The results without the Bonferroni correction are reported in the table in S1 Table (marble run/Blocks) and in the table in S2 Table (Keyboard).

## Results

The characteristics of the sibling interaction were similar for both the play with blocks and the marble run. Consequently, the data of these two play sets were combined. During play with the keyboard, results differed from the other two play sets and are therefore discussed separately.

### Interactive vs. non-interactive behaviour

First, the proportion of time children spent in social interaction (mutuality, interaction with experimenter, interaction with parent) compared to non-social activities (orientation towards sibling, involvement in a purposeful activity) was evaluated. During play with marble run/blocks, LR- and TD-sibs spent on average 15% and 16% of their time in social interaction, respectively. In the HR group, the average proportion of time spent in social interaction was 14% for the HR-sibs and 16% for the ASD-sibs. Differences between the LR and HR group were not significant (younger: *U* = 287.00, *p* = .552; older: *U* = 306.00, *p* = .814). While playing with the keyboard, the average proportion of time spent in social interaction was 15% for LR-sibs, 18% for TD-sibs, 15% for HR-sibs and 21% for ASD-sibs. Again, there were no significant differences between the LR and HR group (younger: *U* = 315.00, *p* = .943; older: *U* = 294.00, *p* = .644). When only looking at social interaction between both siblings (mutuality), the average proportion of time spent in social interaction decreased to 3% for the LR group and 5% for the HR group during play with marble run/blocks, and 3% for the LR group and 2% for the HR group during play with keyboard. Differences between the LR and HR group were all non-significant (marble run/blocks: *U* = 225.00, *p* = .074; keyboard: *U* = 318.50, *p* = .996).

### Characteristics of sibling interactions

Descriptives of the sibling interaction are presented in Table 2. Results of the hierarchical regression models are shown in Table 3 (marble run/blocks) and Table 4 (keyboard).

**Table 2:** Means (standard deviations) of sibling interaction characteristics.

	Marble run and Blocks		Keyboard	
Younger child	LR	HR	LR	HR
Negative initiations^a^	0.63(0.69)	1.09(1.09)	1.48(2.18)	1.27(1.64)
Positive initiations^a^	2.31(2.64)	1.75(1.75)	0.91(1.79)	0.86(1.39)
Negative responses^a^	1.41(1.65)	3.64(2.76)	2.24(2.54)	3.10(4.52)
Positive responses^a^	4.89(4.05)	6.32(4.98)	3.87(3.81)	2.08(2.16)
Mutuality^b^	15.50(16.92)	26.28(27.57)	15.38(21.03)	12.79(15.94)
Orientation to sibling^b^	47.08(18.12)	40.36(34.51)	26.41(21.82)	12.19(13.44)
	Marble run and Blocks		Keyboard	
Older child	LR	HR	LR	HR
Negative initiations^a^	3.57(2.42)	5.05(3.85)	3.86(3.23)	2.68(3.76)
Positive initiations^a^	4.15(4.53)	6.00(8.04)	2.79(4.48)	2.00(2.65)
Negative responses^a^	0.95(1.41)	2.02(2.04)	1.59(2.10)	1.95(2.52)
Positive responses^a^	2.09(2.42)	2.66(3.19)	1.82(2.51)	1.36(1.94)
Mutuality^b^	15.50(16.92)	26.28(27.57)	15.38(21.03)	12.79(15.94)
Orientation to sibling^b^	21.55(27.78)	70.85(122.37)	19.56(22.65)	41.32(60.69)

*Note*. *LR* = low-risk, *HR* = high-risk

^a^results reflect absolute frequencies

^b^results reflect total duration (in seconds)

**Table 3:** Regression coefficients for significant predictors—Marble run/blocks.

	Younger sibling				Older sibling			
		*B (SD)*	β	*R*^*2*^		*B (SD)*	β	*R*^*2*^
Positive initiations	1. (constant)	2.39(.46)		.01	1. (constant)	4.31(1.23)		.05
	Group	-.47(.73)	-.10		Group	2.95(1.95)	.23	
	2. (constant)	3.08(3.79)		.28	2. (constant)	10.19(9.12)		.44^a^
					Age	.11(.03)	.56^a^	
Negative initiations	1. (constant)	.66(.17)		.10	1. (constant)	3.61(.61)		.08
	Group	.59(.27)	.31		Group	1.83(.97)	.28	
	2. (constant)	1.79(1.57)		.20	2. (constant)	1.27(4.43)		.48^a^
					Group	4.96(1.11)	.75^a^	
					SES	.18(.04)	.57^a^	
Positive responses	1. (constant)	5.11(.85)		.07	1. (constant)	2.20(.54)		.03
	Group	2.34(1.34)	.26		Group	1.00(.86)	.17	
	2. (constant)	11.45(6.93)		.33	2. (constant)	3.01(4.30)		.35
					Gender	2.63(.81)	.46^a^	
Negative responses	1. (constant)	1.42(.40)		.19	1. (constant)	.96(.32)		.09
	Group	2.00(.63)	.43		Group	1.01(.50)	.29	
	2. (constant)	.20(3.64)		.28	2. (constant)	.68(2.89)		.19
	Group	3.11(.91)	.68^a^		Group	2.06(.73)	.60^a^	
Orientation to sibling	1. (constant)	46.71(5.40)		.01	1. (constant)	22.20(16.51)		.12
	Group	-6.04(8.55)	-.11		Group	62.49(26.11)	.34	
	2. (constant)	64.78(47.87)		.17	2. (constant)	64.59(110.59)		.57^a^
					Age	2.05(.36)	.78^a^	

*Note*. Gender = gender of the older sibling; Age = age of the older sibling; SES = family SES; Group = high-risk vs. low-risk

^a^remained significant after Bonferroni correction

#### Younger child

During play with marble run/blocks, group was a significant predictor for the amount of negative responses. The model containing both group and the sample characteristics accounted for 28.2% of the variance (*F*(5,39) = 3.071, *p* = .020), but was not significant after the Bonferroni correction. Group, however, was the only remaining significant predictor with more negative responses in the high-risk group. During play with keyboard, group did not significantly predict sibling interaction characteristics after adding the sample characteristics in the model.

**Table 4:** Regression coefficients for significant predictors–keyboard.

	Younger sibling				Older sibling			
		***B (SD)***	**β**	***R***^***2***^		***B (SD)***	**β**	***R***^***2***^
Positive initiations	1. (constant)	.98(.33)		.01	1. (constant)	2.93(.77)		.01
	Group	.02(.52)	.01		Group	-.59(1.22)	-.07	
	2. (constant)	-2.77(3.02)		.10	2. (constant)	10.02(6.55)		.22
Negative initiations	1. (constant)	1.59(.39)		.00	1. (constant)	3.96(.69)		.03
	Group	-.20(.62)	-.05		Group	-1.13(1.09)	-.16	
	2. (constant)	-6.75(3.23)		.26	2. (constant)	.76(6.54)		.05
	DQ	.09(.03)	.50^a^					
Positive responses	1. (constant)	4.01(.64)		.07	1. (constant)	1.96(.45)		.00
	Group	-1.81(1.02)	-.26		Group	-.29(.72)	-.06	
	2. (constant)	9.79(5.87)		.17	2. (constant)	-3.52(3.71)		.29
					Gender	2.27(.70)	.48^a^	
Negative responses	1. (constant)	2.37(.57)		.01	1. (constant)	1.70(.42)		.00
	Group	.41(.91)	.07		Group	.19(.67)	.04	
	2. (constant)	1.66(5.40)		.05	2. (constant)	.91(3.96)		.05
Orientation to sibling	1. (constant)	36.48(3.71)		.12	1. (constant)	20.46(8.54)		.10
	Group	-14.45(5.87)	-.35		Group	28.97(13.51	.31	
	2. (constant)	76.61(31.12)		.34	2. (constant)	27.71(55.19)		.60^a^
					Age	1.04(.18)	.77^a^	
	Sibling pair							
		***B (SD)***	**β**	***R***^***2***^				
Mutuality	1. (constant)	16.52(3.82)		.00				
	Group	-2.31(6.04)	-.06					
	2. (constant)	37.20(33.71)		.16				

*Note*. Gender = gender of the older sibling; Age = age of the older sibling; DQ = developmental quotient younger sibling; Group = high-risk vs. low-risk

^a^remained significant after Bonferroni correction

#### Older child

Play with marble run/blocks revealed two models (including both ‘group’ and the sample characteristics) in which group was a significant predictor. First, the model significantly predicted the *negative initiations*, accounting for 48.3% of the variance (*F*(5,39) = 7.293, *p*<.001). Group was a significant positive predictor with higher levels of negative initiations in the high-risk group. Second, group significantly predicted *negative response*s, meaning that negative responses were more frequent in the high-risk group. However, the overall model was not significant (*R*^2^ = .191, *F*(5,39) = 1.846, *p* = .126). In line with the younger sibling, group did not significantly predict sibling interaction characteristics during play with keyboard.

### Role (a)symmetry

Role (a)symmetry was based upon the number of initiations and responses of both siblings. Higher levels of initiations reflect a more dominant position, while higher levels of responses indicate a more following role. The younger and older child within each group were compared. As in the previous analyses, results for play with the marble run and play with the blocks were combined. Wilcoxon Signed-Rank tests were used to explore within-group differences. Only results that remained significant after the Bonferroni correction are reported.

During play with the marble run/blocks and play with the keyboard, the older sibling in the *LR group* assumed a more dominant position in terms of negative behaviour, reflected in a higher level of negative initiations (marble run/blocks: *z* = -4.49, *p*<.001; keyboard: *z* = -2.97, *p* = .002). In addition, while playing with marble run/blocks the younger siblings followed more frequently, with higher levels of positive responses (marble run/blocks: *z* = -4.26, *p*<.001). In the *HR group*, older children with ASD showed higher levels of negative initiations (*z* = -3.73, *p<*.001) during play with the marble run and blocks, and their younger HR-sibs showed higher levels of positive responses (*z* = -3.15, *p* = .001).

#### Comparison high-risk and low-risk

Finally, it was evaluated whether there was a difference in role (a)symmetry between groups. To determine the degree to which the older sibling was more dominant, the difference score between initiations of the older sibling and initiations of the younger sibling was calculated. To evaluate to which extent the younger sibling was more following, the difference score between responses of the younger sibling and responses of the older sibling was calculated. These difference scores were compared between groups (Mann-Whitney U test). Results revealed no significant difference in the dominance of the older child during play with marble run/blocks (*U* = 219.50, *p* = .058) or keyboard (*U* = 243.50, *p* = .152), or the degree to which the younger child followed (marble run/blocks: *U* = 252.00, *p* = .205; keyboard: *U* = 278.50, *p* = .445).

### Sample characteristics

The degree to which sample characteristics influenced the association between group status (high-risk vs. low-risk) and the sibling interactions was evaluated in the previously mentioned hierarchical regression models (see Tables 3 and 4). Results are discussed in more detail below. Only the results that remained significant after the Bonferroni correction are reported (for results without the correction, see the table in S1 Table and the table in S2 Table).

#### Gender

The gender of the older sibling was a significant predictor for positive responses of the older child during both marble run/blocks and keyboard. When the older sibling was a girl, positive responses (marble run/blocks: β = .455, *p* = .002; keyboard: β = .477, *p* = .002) were more frequent than when the older sibling was a boy.

#### Age

During play with marble run/blocks, the age of the older sibling significantly predicted the positive initiations and orientation to sibling of the older child. The higher the age of the older sibling, the more positive initiations (β = .557, *p* = .001) and orientation to sibling (β = .557, *p* = .001) were observed . During play with keyboard, age of the older sibling only predicted orientation to sibling of the older child, with higher levels of orientation to sibling as the age of the older child increased (β = .771, *p*<.001).

The negative responses of the younger child and negative initiations and responses of the older child during marble run/blocks were still significantly predicted by group status (high-risk vs. low-risk) after adding the sample characteristics in the regression model. However, orientation to sibling of the older child was no longer predicted by group after adding the age of the older child, both during play with the marble run/blocks and play with keyboard.

#### Developmental level younger child

During play with the keyboard, developmental level of the younger child significantly predicted the negative initiations of the younger child. Younger siblings with a lower developmental showed fewer negative initiations (β = .500, *p* = .002)

#### Family SES

During play with marble run/blocks, a higher socioeconomic status was associated with more negative initiations of the older child (β = .567, *p*<.001). Nevertheless, group remained a significant predictor for negative initiations of the older child.

## Discussion

The main focus of this study was to evaluate the characteristics of sibling interactions in dyads involving a child with ASD and its infant sibling in comparison with typically developing dyads. Additionally, we investigated whether the role asymmetry that characterizes early and middle childhood in typically developing dyads was also evident in dyads including a child with ASD. Finally, we explored the potential influence of child characteristics such as gender and age.

The present study revealed similarities as well as differences between dyads including a child with ASD and dyads with only typically developing children. Concerning positive behaviours, there were no clear differences between the HR and LR groups. Both showed a comparable amount of positive initiations and responses in all three play contexts. With regard to negative behaviours, differences were more pronounced. During play with marble run/blocks, HR-sibs and children with ASD showed higher levels of negative behaviours than LR-sibs and typically developing older children, both in terms of negative initiations (ASD-sibs only) and negative responses (ASD-sibs and HR-sibs). Negative initiations included giving a command to or taking a toy from the other sibling, while negative responses included refusing to comply with a request (e.g., giving toy, following command) or counterattacks (e.g., resisting when the other sibling attempted to take a toy away). Play with keyboard did not reveal any group differences. The results are not in agreement with the expectation that children in HR dyads would be less interactive than children in LR dyads. Instead, there was a difference regarding the distinction between positive and negative behaviour.

After exploring the differences in sibling interactions between both groups, we focussed on the role patterns between infants and their older sibling within each group. This has not yet been investigated in a very young age group. During play with marble run/blocks, there was a pattern that older children initiated social interaction more frequently in a negative way (dominant position) while younger children followed and responded more positively. During play with keyboard, the only significant result was an increased level of negative initiations of the older child in the LR group. The comparison of role (a)symmetry between groups revealed no significant differences. Thus, against our expectations, the role asymmetry (during play with marble run/blocks) was also found in HR dyads.

Our findings in the HR group are not in line with the findings of Knott and colleagues [40,47] or Walton and Ingersoll [48]. In addition, with regard to role (a)symmetry, Knott and colleagues [40,47] found that the younger typically developing child took over the dominant position of the older child with ASD, while this was not the case in our study. It must be noted, however, that the children in the studies of Knott et al. [40,47] and Walton and Ingersoll [48] were on average older than the children in this study. Consequently, differences in results could be related to the younger sample. First, the level of social interaction in children and their *interest* in other interaction partners partially depends on the age of the child. In the present study the overall level of mutual interaction between the 18-month-old infants and their older sibling was low in the LR as well as in the HR group (5% or less of the total interaction). Previous research by Lamb [1] also emphasized the limited amount of direct interaction between young siblings. In their study, younger infants and preschool-aged children showed more interest in interacting with their parent than with their sibling. Moreover, siblings stayed close to each other and often played with the same toys, but they were rather engaged in parallel than in mutual play. Second, the child’s age determines the level of social-communicative development and the extent to which children are *capable* of participating in and/or leading social interactions. Infants will show fewer social-communicative abilities than toddlers/pre-schoolers or school-aged children. In addition, even though 18-month-old infants already display levels of simple social play during peer interactions, more complex forms of social play are not yet developed [72]. In sum, younger children could be less motivated and/or could lack the social-communicative abilities needed to participate in social interaction as well as to take on a dominant/leading position. Therefore, the young age of the current sample could explain the lower overall level of interaction, the limited group differences and the discrepancy between the current study and the studies of Knott at al. [40,47] and Walton and Ingersoll [48].

The limited time younger sibling pairs spend in mutual interaction and infants’ lower levels of social-communicative abilities reduce the possibility of finding differences between ASD dyads and typically developing dyads. Nevertheless, the current study found significant group results, confirming that this younger age group should not be neglected. Especially the presence of negative behaviour in the HR group is noteworthy, possibly disturbing the balance between positive and negative interactions. This may impact on the development of HR-sibs. On the one hand, previous research has shown that sibling relationships high in conflict are associated with lower levels of social competence and self-worth, and higher levels of internalizing and externalizing problems [9]. On the other hand, when combined with warmth and nurturing interactions, conflict can lead to a more positive outcome [10]. Participating in conflict rather than submitting to negative behaviour of the other sibling can benefit children’s development, for example by promoting their problem-solving abilities.

As a result of the social-communicative difficulties associated with ASD, it can be expected that children with ASD experience difficulties in adequately initiating social interaction with a younger sibling or responding appropriately to their siblings’ social approaches, resulting in higher levels of negative initiations and responses. Children with ASD also show higher rates of aggressive behaviour, depending on their cognitive functioning and language development [73–75]. While negative behaviour in children with ASD can be associated with characteristics of the disorder, the origin of higher levels of negative behaviour in HR-sibs is less clear. First, through bidirectional processes, HR-sibs’ responses may be influenced by repeated confrontation with the social-communicative difficulties of the child with ASD. For example, higher amounts of negative initiations or responses in children with ASD could trigger more negative responses in the HR-sib. After a while, this might become a learned interaction style. A second possibility is observational learning. Siblings learn and develop by observing and imitating one another, both in terms of positive (learning social competencies) and negative (conflict, hostility, aggression) behaviours [43,44]. Thus, HR-sibs might imitate the more negative behaviours from their older brother/sister with ASD. Third, genetic factors can lead to (subclinical) characteristics of ASD in HR-sibs as well, leading to more negative behaviours during social interaction.

It is noteworthy that the group differences changed as a function of the play materials. One possible explanation lies in the type of interactions that the play materials trigger. Since there was only one keyboard (i.e., limited resources), the risk of conflict during play with keyboard was higher than during play with marble run/blocks. Accordingly, siblings in the LR group indeed showed higher levels of negative initiations during keyboard than during marble run/blocks. However, this was not the case in the HR group. It is possible that, while the LR group only showed an increase in negative behaviour as a response to the limited resources, levels of negative behaviour in the HR group were higher regardless of the play set. As a result, the difference in negative behaviour between both groups was significant during play with marble run/blocks, but not during keyboard (since both groups display higher levels of negative behaviour).

This study has some limitations that need to be acknowledged. Although significant findings were observed, the small sample size reduced the power of the study and the likelihood of detecting significant results. After applying a Bonferroni correction, several significant results were no longer significant. This is possibly due to a decrease in power and does not necessarily mean that there are no real world differences. Therefore, results need to be interpreted with caution.

Second, parametric analyses, based on a larger sample size, would have allowed for more elaborate analyses such as evaluating the possible influence of sample characteristics. In the current study, the LR and HR group were not matched in terms of several sample characteristics. Although we cannot completely exclude the possibility that sample characteristics affected our findings, results from the hierarchical regression analyses and additional correlational analyses provide relevant insights into the association between these sample characteristics and sibling interaction variables. With regard to the negative behaviours, results from the regression analyses show that group differences remained significant after adding sample characteristics into the model. This suggests that the group differences in terms of negative behaviours might reflect genuine group differences between the LR and HR group. Concerning positive behaviours, which were more likely to occur in dyads with an older sister regardless of group status, the lack of differences between the LR and HR group could be caused by group differences in terms of gender. Unfortunately, due to the limited number of girls with ASD, we could not compare HR sibling pairs with an older sister with HR sibling pairs with an older brother and evaluate gender differences within the HR group. Gender differences in sibling relationships should be further evaluated in a larger sample, including a higher number of girls with ASD. Associations between the sibling interaction and characteristics such as developmental level of the youngest sibling, family SES and age of the older sibling were limited, giving little indication that these significantly impacted on the found group differences. In addition, the average mental age of the children with ASD was somewhat lower than their chronological age, reducing the gap with typically developing older children.

Even though a matched control group is necessary to evaluate the role of sample characteristics, we also need to take into account that these differences in sample characteristics could be inherent to the ASD population. Boys are more frequently diagnosed with ASD than girls [76]. In addition, raising a child with ASD is associated with lower feelings of parenting efficacy, lower parental well-being, financial strain, etc. [77]. Combined with the increased recurrence risk in siblings of children with ASD [24], this could lead to a larger time interval between children with ASD and later-born siblings and a larger age gap between a child with ASD and a younger sibling. Thus, in daily life, HR-sibs’ sibling interactions are more likely to include older brothers with ASD. To avoid this sampling bias in empirical studies, a carefully selected control group is needed, matched in terms of relevant sample characteristics (age, gender, SES,…). Unfortunately, because the current study was part of a longitudinal follow-up study of HR-sibs and a LR control group who were not selected based on gender and age of the older sibling, we were unable to match the groups in terms of these sample characteristics.

We also need to take into consideration that LR-sibs attended day care more frequently than HR-sibs, meaning that LR-sibs had more social experiences than HR-sibs. These experiences could improve the LR-sibs’ social-communicative abilities, which could in turn influence their behaviour during sibling interaction. ASD severity and cognitive functioning of the older child were not added in the regression models because of the small sample size and the lack of comparability between measures (IQ). However, given their possible influence on the results, correlational analyses were performed to evaluate the association with the sibling interaction. The association between cognitive functioning and the sibling interaction characteristics (i.e., positive/negative initiations/responses) in the HR group was limited, with only significant positive associations between the ASD-sib’s cognitive functioning/IQ and 1) positive initiations of the older child during play with marble run/blocks, 2) negative initiations of the younger child during play with keyboard, and 3) positive responses of the older child during play with keyboard. However, these associations were no longer significant after applying a Bonferroni correction. Spearman’s rank correlation coefficients are presented in the table in S3 Table. Finally, ASD severity of the older child (i.e., SCQ and SRS scores) was not significantly correlated with positive/negative initiations or responses during both play contexts.

Future research should focus on replicating the current results in a larger sample, including a LR control group matched in terms of relevant sample characteristics. Second, not only younger siblings’ development is affected by the presence of a child with ASD in the family. The development of older siblings of children with ASD should be considered as well. Third, the relationship between sibling interactions and child outcome could be influenced by social interactions with other family members. Due to practical reasons, the current study was unable to include sibling interactions between HR-sibs and an older sibling without ASD. Finally, the children were too young for diagnostic assessment. Therefore we were unable to evaluate differences between typically developing HR-sibs and HR-sibs with characteristics of BAP or ASD.

The results of the current study raise theoretical implications. While the development of HR-sibs is increasingly being studied, the current study is the first to consider sibling interactions between very young HR-sibs and their older sibling with ASD as a possible link between early vulnerabilities and later outcome. Even with the overall level of interaction being rather low, the sibling interactions in the HR group were clearly more negative than in the LR group. Altered early sibling interactions in terms of higher levels of negative behaviour could influence the learning environment of young HR-sibs, possibly changing their early developmental trajectory [39,78]. If the higher level of negative initiations and responses disturbs the balance between positive and negative, this could lead to higher levels of internalizing or externalizing problems and lower social competence in both children [9]. Although conflict and conflict resolution can benefit development as well (e.g., [28]), negative behaviours in the current study are not always related to conflict (e.g., taking something from the sibling). Longitudinal studies could provide further evidence on the pathway between early sibling interactions and child outcome. For example, in their longitudinal study Wan et al. [79] provided support for the association between early parent-child interactions at 12 months and ASD outcome at 36 months.

Second, if indeed differences in early sibling interactions lead to differences in developmental trajectories of HR-sibs, sibling interactions should be targeted in early interventions in ASD, taking into account the development of both siblings. In this way, interventions can promote positive sibling relationships and individual adjustment of both siblings [5]. Interventions that target those aspects of sibling interactions that negatively influence child development, could improve the later outcome of both HR-sibs and children with ASD. Nevertheless, sibling interactions should be considered within the entire family system, including the interaction with other family members (e.g., parents). Targeting sibling interactions could be part of a broader intervention or could be included in specific programs such as home guidance.

Despite the limitations, this study provides new insights in the early learning environment of HR-sibs. To date, the existing research on sibling relationships in families with a child with ASD is limited, especially including siblings as young as 18 months. The present study demonstrates that, by using naturalistic observations (i.e., observing behaviour in a natural setting), differences in early sibling interactions between ASD dyads and typically developing dyads can be detected. Further research is required to determine the impact of these sibling interactions on child development and family functioning, and to assess whether higher levels of negativity support or compromise the development of HR-sibs. This could in turn provide insights on the potential value of sibling interactions in early intervention.

## Supporting information

S1 Table Regression coefficients for significant predictors with and without the Bonferroni correction—Marble run/blocks. (DOCX)

S2 Table Regression coefficients for significant predictors with and without the Bonferroni correction–keyboard. (DOCX)

S3 Table Correlation (Spearman's rank correlation coefficients) between the cognitive functioning of the older child with ASD and sibling interaction characteristics of both children. (DOCX)
