# Supplementary material for: The early development of infant siblings of children with autism spectrum disorder: Characteristics of sibling interactions
Source: PLoS One. 2018 Mar 15;13(3):e0193367. doi: 10.1371/journal.pone.0193367 (PMC5854306; doi:10.1371/journal.pone.0193367)
Supplement: S1 Table — (DOCX) [file pone.0193367.s001.docx]

# Supporting information

| **Regression coefficients for significant predictors with and without the Bonferroni correction - Marble run/Blocks** | | | | | | | | | |
| --- | --- | --- | --- | --- | --- | --- | --- | --- | --- |
|  | Younger sibling | | | |  | Older sibling | | | |
|  |  | ***B (SD)*** | **β** | ***R²*** |  |  | ***B (SD)*** | **β** | ***R²*** |
| Positive initiations | 1. (constant) | 2.39(.46) |  | .01 |  | 1. (constant) | 4.31(1.23) |  | .05 |
|  | Group | -.47(.73) | -.10 |  |  | Group | 2.95(1.95) | .23 |  |
|  | 2. (constant) | 3.08(3.79) |  | .28* |  | 2. (constant) | 10.19(9.12) |  | .44***^a^ |
|  | Gender | 1.85(.71) | .38* |  |  | Gender | 4.67(1.72) | .35** |  |
|  | Age | .03(.01) | .36^+^ |  |  | Age | .11(.03) | .56*** ^a^ |  |
|  |  |  |  |  |  |  |  |  |  |
| Negative initiations | 1. (constant) | .66(.17) |  | .10* |  | 1. (constant) | 3.61(.61) |  | .08 |
|  | Group | .59(.27) | .31* |  |  | Group | 1.83(.97) | .28 |  |
|  | 2. (constant) | 1.79(1.57) |  | .20 |  | 2. (constant) | 1.27(4.43) |  | .48***^a^ |
|  | Group | .98(.40) | .52* |  |  | Group | 4.96(1.11) | .75***^a^ |  |
|  | Age | -.01(.01) | -.40* |  |  | Age | -.04(.01) | -.44** |  |
|  |  |  |  |  |  | SES | .18(.04) | .57***^a^ |  |
|  |  |  |  |  |  |  |  |  |  |
| Positive responses | 1. (constant) | 5.11(.85) |  | .07 |  | 1. (constant) | 2.20(.54) |  | .03 |
|  | Group | 2.34(1.34) | .26 |  |  | Group | 1.00(.86) | .17 |  |
|  | 2. (constant) | 11.45(6.93) |  | .33* |  | 2. (constant) | 3.01(4.30) |  | .35** |
|  | Gender | 2.74(1.30) | .30* |  |  | Gender | 2.63(.81) | .46**^a^ |  |
|  | Age | .05(.02) | .38* |  |  | Age | .03(.01) | .37* |  |
|  | DQ | -.12(.06) | -.29* |  |  |  |  |  |  |
|  |  |  |  |  |  |  |  |  |  |
| Negative responses | 1. (constant) | 1.42(.40) |  | .19** |  | 1. (constant) | .96(.32) |  | .09 |
|  | Group | 2.00(.63) | .43** |  |  | Group | 1.01(.50) | .29 |  |
|  | 2. (constant) | .20(3.64) |  | .28* |  | 2. (constant) | .68(2.89) |  | .19 |
|  | Group | 3.11(.91) | .68**^a^ |  |  | Group | 2.06(.73) | .60**^a^ |  |
|  |  |  |  |  |  | Age | -.02(.01) | -.40* |  |
|  |  |  |  |  |  |  |  |  |  |
| Orientation to sibling | 1. (constant) | 46.71(5.40) |  | .01 |  | 1. (constant) | 22.20(16.51) |  | .12* |
|  | Group | -6.04(8.55) | -.11 |  |  | Group | 62.49(26.11) | .34* |  |
|  | 2. (constant) | 64.78(47.87) |  | .17 |  | 2. (constant) | 64.59(110.59) |  | .57***^a^ |
|  | Age | -.37(.16) | -.46* |  |  | Age | 2.05(.36) | .78***^a^ |  |
| *Note.* ^+^p=.053,**p*<.05, ***p*<.01, ****p*<.001; Gender = gender of the older sibling; Age = age of the older sibling; DQ = developmental quotient younger sibling; SES = family SES; Group = high-risk vs. low-risk; ^a^remained significant after Bonferroni correction | | | | | | | | | |
