# Supplementary material for: The early development of infant siblings of children with autism spectrum disorder: Characteristics of sibling interactions
Source: PLoS One. 2018 Mar 15;13(3):e0193367. doi: 10.1371/journal.pone.0193367 (PMC5854306; doi:10.1371/journal.pone.0193367)
Supplement: S2 Table — (DOCX) [file pone.0193367.s002.docx]

# Supporting information

| **Regression coefficients for significant predictors with and without the Bonferroni correction - Keyboard** | | | | | | | | | |
| --- | --- | --- | --- | --- | --- | --- | --- | --- | --- |
|  | Younger sibling | | | |  | Older sibling | | | |
|  |  | ***B (SD)*** | **β** | ***R²*** |  |  | ***B (SD)*** | **β** | ***R²*** |
| Positive initiations | 1. (constant) | .98(.33) |  | .01 |  | 1. (constant) | 2.93(.77) |  | .01 |
|  | Group | .02(.52) | .01 |  |  | Group | -.59(1.22) | -.07 |  |
|  | 2. (constant) | -2.77(3.02) |  | .10 |  | 2. (constant) | 10.02(6.55) |  | .22 |
|  |  |  |  |  |  | Gender | 3.22(1.23) | .40* |  |
|  |  |  |  |  |  |  |  |  |  |
| Negative initiations | 1. (constant) | 1.59(.39) |  | .00 |  | 1. (constant) | 3.96(.69) |  | .03 |
|  | Group | -.20(.62) | -.05 |  |  | Group | -1.13(1.09) | -.16 |  |
|  | 2. (constant) | -6.75(3.23) |  | .26* |  | 2. (constant) | .76(6.54) |  | .05 |
|  | DQ | .09(.03) | .50**^a^ |  |  |  |  |  |  |
|  |  |  |  |  |  |  |  |  |  |
| Positive responses | 1. (constant) | 4.01(.64) |  | .07 |  | 1. (constant) | 1.96(.45) |  | .00 |
|  | Group | -1.81(1.02) | -.26 |  |  | Group | -.29(.72) | -.06 |  |
|  | 2. (constant) | 9.79(5.87) |  | .17 |  | 2. (constant) | -3.52(3.71) |  | .29* |
|  |  |  |  |  |  | Gender | 2.27(.70) | .48**^a^ |  |
|  |  |  |  |  |  |  |  |  |  |
| Negative responses | 1. (constant) | 2.37(.57) |  | .01 |  | 1. (constant) | 1.70(.42) |  | .00 |
|  | Group | .41(.91) | .07 |  |  | Group | .19(.67) | .04 |  |
|  | 2. (constant) | 1.66(5.40) |  | .05 |  | 2. (constant) | .91(3.96) |  | .05 |
|  |  |  |  |  |  |  |  |  |  |
| Orientation  to sibling | 1. (constant) | 36.48(3.71) |  | .12* |  | 1. (constant) | 20.46(8.54) |  | .10* |
|  | Group | -14.45(5.87) | -.35* |  |  | Group | 28.97(13.51 | .31* |  |
|  | 2. (constant) | 76.61(31.12) |  | .34** |  | 2. (constant) | 27.71(55.19) |  | .60***^a^ |
|  | Gender | 13.06(5.85) | .31* |  |  | Age | 1.04(.18) | .77***^a^ |  |
|  | DQ | -.60(.26) | -.32* |  |  | SES | -1.23(.52) | -.28* |  |
|  | Sibling pair | | | |  |  |  |  |  |
|  |  | ***B (SD)*** | **β** | ***R²*** |  |  |  |  |  |
| Mutuality | 1. (constant) | 16.52(3.82) |  | .00 |  |  |  |  |  |
|  | Group | -2.31(6.04) | -.06 |  |  |  |  |  |  |
|  | 2. (constant) | 37.20(33.71) |  | .16 |  |  |  |  |  |
|  | Gender | 14.42(6.34) | .36* |  |  |  |  |  |  |
| *Note.* **p*<.05, ***p*<.01, ****p*<.001; Gender = gender of the older sibling; Age = age of the older sibling; DQ = developmental quotient younger sibling; SES = family SES; Group = high-risk vs. low-risk; ^a^remained significant after Bonferroni correction | | | | | | | | | |
