# Supplementary material for: The early development of infant siblings of children with autism spectrum disorder: Characteristics of sibling interactions
Source: PLoS One. 2018 Mar 15;13(3):e0193367. doi: 10.1371/journal.pone.0193367 (PMC5854306; doi:10.1371/journal.pone.0193367)
Supplement: S3 Table — (DOCX) [file pone.0193367.s003.docx]

# Supporting information

| **Correlation (Spearman's rank correlation coefficients) between the cognitive functioning of the older child with ASD and sibling interaction characteristics of both children** | | | | |
| --- | --- | --- | --- | --- |
|  | **Marble run/blocks** | | | |
|  | Younger child | | | |
|  | Negative Initiations | Positive Initiations | Negative  Responses | Positive  Responses |
| Cognitive functioning | .25 | .37 | -.11 | .38 |
|  | Older child | | | |
|  | Negative Initiations | Positive Initiations | Negative  Responses | Positive  Responses |
| Cognitive functioning | -.09 | .46* | -.07 | .43 |
|  |  |  |  |  |
|  | **Keyboard** | | | |
|  | Younger child | | | |
|  | Negative Initiations | Positive Initiations | Negative responses | Positive  Responses |
| Cognitive functioning | .46* | .23 | .39 | -.02 |
|  | Older child | | | |
|  | Negative Initiations | Positive Initiations | Negative Responses | Positive  Responses |
| Cognitive functioning | .08 | .24 | .35 | .46* |
| *Note.* **p*<.05; Cognitive functioning/IQ of the older child with ASD divided into five categories (<70, 70-85, 85-115, 115-130, >130) | | | | |
